# Histopathological Profiles of Rats (*Rattus norvegicus*) Induced with Streptozotocin and Treated with Aqueous Root Extracts of *Ruellia tuberosa* L.

**DOI:** 10.1155/2021/6938433

**Published:** 2021-11-10

**Authors:** Anna Safitri, Dewi Ratih Tirto Sari, Bigram Refsilangi, Anna Roosdiana, Fatchiyah Fatchiyah

**Affiliations:** ^1^Chemistry Department, Brawijaya University, Malang, Indonesia; ^2^Research Centre of Natural Genetic Resources (SMONAGENES), Brawijaya University, Malang, Indonesia; ^3^Biology Department, Brawijaya University, Malang, Indonesia

## Abstract

Diabetes mellitus (DM) is a serious worldwide health threat since the number of people with DM is forecasted to grow annually. Thus, effective and affordable treatment is urgently needed. Our previous studies used n-hexane and hydroethanolic root extracts of *Ruellia tuberosa* L. which significantly affected diabetic rats. In this study, aqueous *R. tuberosa* L. root extracts were used as treatments for the diabetic rat model and their effects were evaluated. Diabetes was generated by the administration of streptozotocin (STZ) at 20 mg/kg within 5 sequential days. Male Wistar rats were orally treated with the extracts and standard drug (metformin 200 mg/kg) and vehicle every day for 4 weeks. Hypoglycemic effects were assessed for normal, diabetic control, standard, and extract-treated groups. Histopathology was also carried out for the pancreatic, hepatic, and kidney tissues. The progression of diabetes was considerably diminished after extract treatment. In treated rats, the highest dose of extract induced a decline in blood glucose and serum malondialdehyde (MDA) levels at 25% and 35%, respectively. Furthermore, aqueous extract of *R. tuberosa* L. treatment decreased MDA levels in the pancreas by 12%. Histologic examination of the organ tissues of diabetic rats showed deteriorating alterations. After treatment, histopathological damages to the tissues and cells were reversed. The results of the experiments recommend that aqueous extract of *R. tuberosa* L. has antidiabetic effects on STZ-induced diabetic rats; nevertheless, a higher dose of the aqueous extracts is needed to achieve more significant results.

## 1. Introduction

Diabetes mellitus is classified as a metabolic disorder owing to daily-life problems in the 21st century [1]. In the pathophysiology of diabetes, insulin, the hormone that is produced in pancreatic *ß*-cells, is essential [2]. The occurrence of diabetes has reached epidemic magnitudes and is constantly growing globally. The number of people with diabetes is estimated to be two-fold, from 171 million to 366 million people by 2020 [3]. A comparable trend is seen in Indonesia. Statistics from the Ministry of Health of the Republic of Indonesia affirm that, in 2006, there were just about 8.4 million people in Indonesia who suffered from diabetes [4]. By 2030, this figure is forecasted to grow to 21.3 million people [4]. Diabetes mellitus management is mostly managed by the administration of insulin and antidiabetic oral drug consumption [5]. Nevertheless, these approaches involve huge budgets and are at risk of causing unpleasant adverse consequences. Because DM treatments are costly, physicians and investigators begin to explore substitute remedies from natural products that are reasonable and have minimum side effects associated with medication using chemical substances.

Exploring bioactive compounds from natural materials can contribute to knowledge in the health sector, both in preventing and treating disease. Indonesia has a very abundant diversity of plants that can be utilized to supply raw ingredients for traditional medicine. One of the plants that can be used as a resource of bioactive compounds is from the Acanthaceae family [6]. The genus *Ruellia* is one of the members of the Acanthaceae family. *Ruellia tuberosa* L. is broadly spread in Asian countries, i.e., Indonesia [6]. In traditional remedies, this flora is recognized as an antidiuretic, antidiabetic, analgesic, and antihypertensive [7–9].

Based on our previous studies, n-hexane root extracts from *R. tuberosa* L. have activity as an antidiabetic drug in rat diabetic models [10]. These results were shown by blood glucose reduction, MDA level decrease, and kidney histopathological profile recovery [10]. A qualitative test has demonstrated that triterpenoid compounds were present in the n-hexane *R. tuberosa* L. root extracts. Subsequently, an in vivo study of *R. tuberosa* L. hydroethanolic root extracts was conducted and discovered that the extracts had effects on the decrease of blood glucose and MDA levels and affected serum enzyme activity [11–13]. The phytochemical examination carried out in this study found that phytosterol, phenolic, and flavonoid compounds were enclosed in *R. tuberosa* L. hydroethanolic root extracts [11]. The Fourier-transform infrared (FTIR) spectroscopy and LC-MS (liquid chromatography-mass spectrometry) studies on the kidney histopathological profiles, kidney MDA concentrations, and TNF (tumor necrosis factor) alpha expression of the kidney from diabetic rats after being treated with hydroethanolic root extracts of *R. tuberosa* L. have been reported [14, 15].

Many secondary metabolites, such as flavonoids, and other phenolic compounds commonly dissolve in the polar solvent [16]. Therefore, in this study, a single solvent system, i.e., water, is used to extract the roots of *R. tuberosa* L. The aqueous root extracts of *R. tuberosa* L. are suggested to be benign and efficient which can be used as a natural remedy.

The compounds in the aqueous root extracts of *R. tuberosa* L. were identified and characterized previously [17]. In the antioxidant test using DPPH (*α*, *α*-diphenyl-*β*-picrylhydrazyl) free radicals, the aqueous root extracts resulted in an IC_50_ value of 15.2 mg/ml [17]. The antidiabetic capacity of the aqueous root extracts was established by performing an in silico study of the extract compounds. Results concluded that the aqueous root extracts of *R. tuberosa* L. have the potential to be used as antidiabetic agents [17]. Considering the biological activities of *R. tuberosa* L. that have been shown previously, it is interesting to evaluate the pharmacological activities of the antidiabetic effect of the aqueous root extracts of *R. tuberosa* L. In this work, the hypoglycemic and histopathological profiles from treatments to diabetic rats using the aqueous root extracts of *R. tuberosa L*. are investigated.

## 2. Materials and Methods

### 2.1. Plant Material

The dried root powder of *R. tuberosa* L. was obtained from Unit Pelaksana Teknis (UPT) or technical operation unit Materia Medica Batu, East Java, Indonesia. The plant was authenticated by a taxonomist from UPT Materia Medica and was accompanied by a letter of confirmation of the species name.

### 2.2. Total Phenolic and Flavonoid Content of the Extracts

The total phenolic content of the extract was determined using the Folin–Ciocalteu reagent, following the method explained by Singleton and Rossi [18] with some alterations. An aliquot of the sample solution was diluted with water (4x), and Folin–Ciocalteu reagent was added (1 : 1). 1 mL of saturated sodium carbonate solution (8% w/v in water) was added to the solution mixture. The mixture was left at room temperature for 30 min in a dark condition. The absorbance of the solution was measured at 765 nm with a UV-Vis spectrophotometer (1601, Shimadzu). Total phenolic content was expressed as gallic acid equivalent (GAE/g of sample) based on a standard curve of gallic acid (5–500 mg/L). All measurements were conducted in triplicates.

Quercetin was used as a reference compound for total flavonoid determination. A quercetin stock solution was formulated by dissolving 5.0 mg of quercetin in 1.0 mL of methanol; then, a standard solution of quercetin was set by serial dilution using methanol (5–200 µg/mL). An aliquot of quercetin solution or extract solution was individually mixed with 2% aluminium chloride (1 : 1). After stirring together, the solution was set aside at room temperature for 60 min. The absorbance of the reaction mixture was determined at 420 nm with a UV-Vis spectrophotometer (1601, Shimadzu). The total concentration of flavonoid was calculated from a standard curve and stated as mg quercetin equivalent (QE)/g. All measurements were conducted in triplicates.

### 2.3. FTIR Spectroscopic Analysis of Functional Groups of the Extracts

The aqueous extracts of *R. tuberosa* L. were identified as their functional groups using a Fourier-transform infrared resonance (FTIR) spectrometer (1600S, Shimadzu). The measurements were performed at room temperature and recorded from 4000 to 400 cm^−1^ wavenumber with a spectral resolution of turnover of 4 cm^−1^. The frequencies of wavenumbers were assigned to the reference literature [19, 20] to identify the functional groups that exist in the aqueous root extract of *R. tuberosa* L.

### 2.4. Diabetes Induction and Treatment

This in vivo study was officially permitted by the Ethics Committee of Brawijaya University with letter no. 1124-KEP-UB. Preparation of the aqueous extracts was previously reported in [17]. Briefly, 20 albino male Wistar rats (7-8 weeks old and body weight 120–180 g) were obtained from the Biosains Institute, Brawijaya University. As soon as the rats arrived, they were adapted to individual confined cages for 1 week with an ambient temperature (22 ± 3 °C) and light rotation of 12 h/12 h light-dark. The rats were fed with a standard diet and water ad libitum. After the adaptation period, the rats were separated into five groups (*n* = 4 in each group): control (group 1); diabetic (group 2); and diabetic treated with 250 mg/kg extracts (group 3), 500 mg/kg extracts (group 4), and 200 mg/kg metformin (group 5). The extract doses were selected based on the doses used in our previous study [15]. Diabetes mellitus was induced by streptozotocin (STZ) i.p administration with a low dose of 20 mg/kg for five consecutive days. Aqueous extracts and metformin were orally given daily for 4 weeks. The medium-term treatment was applied based on our previous studies on *R. tuberosa* L. on in vivo studies [10, 15]. Blood glucose levels of overnight-fasted rats were measured every week. In the last week of the experimental time, the rats were fasted for the night; rat organs and blood were harvested for several biochemical examinations.

### 2.5. Histopathological Examination

After the termination of the experiments, the pancreas, livers, and kidneys were examined for histopathological studies. Histological procedures were based on methods described earlier in [19]. These organs were cut into thin-sectioned pieces (1 mm × 1 mm × 1 mm). The sections were paraffin-embedded by standard techniques and were stained with haematoxylin and eosin. All organs were then carefully investigated under a microscope (SZX16 Olympus) using 600 × magnification.

### 2.6. Biochemical Determination

Serum was obtained by centrifugation of the blood samples at 3000 g for 10 min in a cold microcentrifuge. Then, MDA concentration in serum was determined following the procedures as in [15] with slight modifications. The MDA concentration was calculated using a QuantiChrom™ TBARS assay kit. The procedures were conducted according to manufacturer's instructions. A UV-Vis spectrophotometer (Shimadzu UV-visible spectrophotometer UV-1601) was used to detect the intensity of the colour changes at the 530 nm wavelength on the samples, after the TBA (thiobarbituric acid) reagent was added. The MDA concentration in the pancreas was also determined by a method similar to the abovementioned method. The absorbance values were compared to the MDA levels in the samples.

### 2.7. Statistical Analysis

Results were stated as means ± standard error. The statistical package for Social Science (SPSS) version 23.0 for Windows was used for the statistical analyses. The one-way analysis of variance (ANOVA) followed by Tukey's post hoc test was used for the analysis of data. The significant differences were set at *p* < 0.05. Histopathological profiles of the organs were analyzed qualitatively.

## 3. Results

### 3.1. Identification of Aqueous Root Extract of *R. tuberosa* L. Metabolites Using FTIR

The total phenolic content of the aqueous *R. tuberosa* L. extract was obtained at 3619.231 ± 0.29 mg GAE/L extract. The total flavonoid content of the extract was 1062.121 ± 0.25 mg QE/L extract. These results show that the aqueous extract of the root contains flavonoids and phenolic compounds.

**Figure 1:**
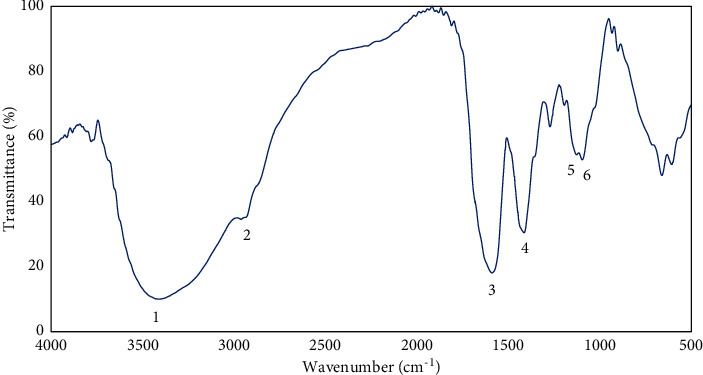
FTIR spectra of the aqueous root extract of *R. tuberosa* L.

The infrared spectroscopic study of the FTIR spectra in the midinfrared region (4000–400 cm^−1^) for the aqueous extract of *R. tuberosa* L. is shown in Figure 1, and characteristic peaks seen in the spectra were numbered 1 to 6. Number 1 to 6 peaks were as follows: 3408.75 cm^−1^; 2963.22 cm^−1^; 1588.07 cm^−1^; 1414.49 cm^−1^; 1127.11 cm^−1^; and 1096.25 cm^−1^. Those peaks were identified as alcohol O–H stretching, C–H aliphatic, C = C aromatics, C–C aromatic, C–O alcohol, and C–H from the aromatic nucleus, for peak numbers 1 to 6, respectively [20, 21]. From these spectrum assignments, the secondary metabolite compounds in the extract were mostly flavonoids or phenolic compounds.

### 3.2. Hypoglycemic Activity of the Aqueous Root Extract of *R. tuberosa* L

The effects of the aqueous root extracts of *R. tuberosa* L. on blood glucose levels of rats are listed in Table 1. The elevated glucose levels in the diabetic group were discovered to be highly significant, 299.24% compared to that of the normal group. Blood glucose levels declined after oral administration of the extracts (250 and 500 mg/kg) or metformin (200 mg/kg). Nonetheless, diabetes treatments with the aqueous root extracts of *R. tuberosa* L. at the highest dose resulted in a 25.35% decrease. In the metformin treatment group, the blood glucose declined up to 68.42%.

Another hypoglycemic activity of the aqueous root extracts of *R. tuberosa* L. can be observed from decreases in MDA levels. The results of the MDA concentration measurements are shown in Table 2. In the untreated diabetic group, the MDA concentrations mounted up to 448% and 216.5% in the serum and pancreas, respectively. After the treatment, MDA levels in treated groups (groups 3, 4, and 5) decreased significantly (at *p* < 0.05). The maximum dose used (500 mg/kg body weight) resulted in higher decreases in the MDA levels in the pancreas and serum at 12% and 34.6%, respectively.

Histopathological profiles from treatments to diabetic rats with the aqueous root extracts of *R. tuberosa* L.

**Figure 2:**
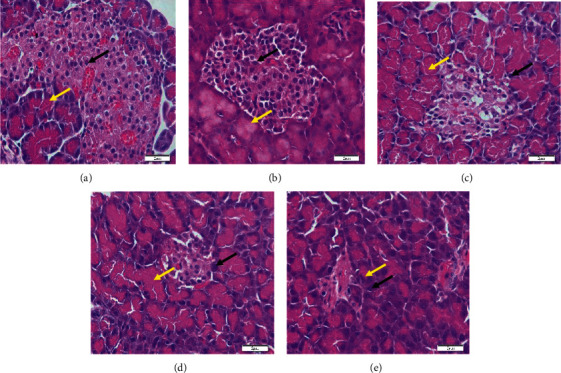
Histopathology of the pancreas in (a) the normal control group; (b) the diabetic control group; (c) diabetic rats treated with 250 mg/kg extracts; (d) diabetic rats treated with 500 mg/kg extracts; and (e) diabetic rats treated with metformin 200 mg/kg, with 600× magnification. Black arrows show beta cells; yellow arrows show Langerhans islet.

**Figure 3:**
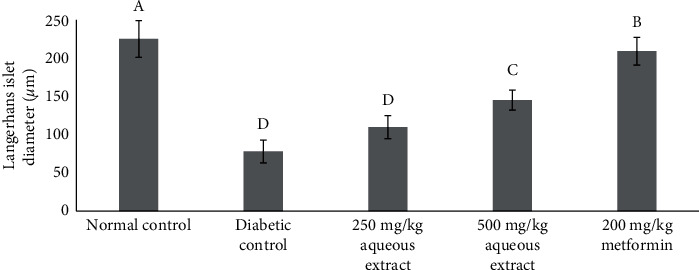
Langerhans islet diameters for all groups. Different notations indicate significant differences between treatments (*p* < 0.05).

Figure 2(a) shows an islet of Langerhans of Wistar rats in the normal control group. The islet shows a high number of beta cells dispersed all through the islet. In the diabetic group, a fall in the number of beta cells was observed as compared to that in the normal control group rats (Figure 2(b). The degeneration of the beta cells was caused by the streptozotocin used to trigger diabetes. Histopathology of the extract-treated groups shows the partial repair of islets of Langerhans, as shown in Figures 2(c), 2(d), and 2(e). The improvement of necrotic beta cells was remarkably more distinct after treatment with 200 mg/kg metformin. In untreated diabetic rats, a significant reduction in the diameter of islet cells was observed, as shown in Figure 3. Treatments with 500 mg/kg aqueous extract have significantly increased the reduction of Langerhans islet diameter. Treatments with 200 mg/kg metformin have a more significant reduction of the islet cell diameter. A smaller dose of aqueous extract (250 mg/kg), however, cannot increase the diameter of the islet cells.

**Figure 4:**
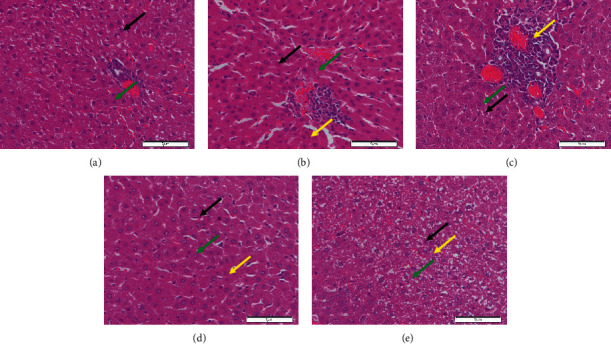
Histopathology of the liver in (a) the normal control group; (b) the diabetic control group; (c) diabetic rats treated with 250 mg/kg extracts; (d) diabetic rats treated with 500 mg/kg extracts; and (e) diabetic rats treated with metformin 200 mg/kg, with 600 × magnification. Black arrows show hepatocytes; yellow arrows show vacuolization; and green arrows show the sinusoidal layer.

**Figure 5:**
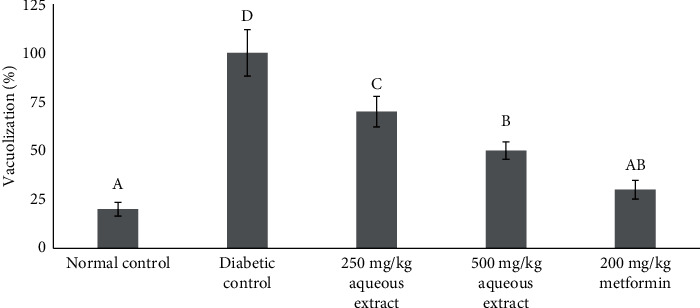
Vacuolization or lipid droplets for all groups. Different notations indicate significant differences between treatments (*p* < 0.05).

Liver segments from the normal control group, as shown in Figure 4(a), revealed normal hepatic features characterized histologically as the numerous hepatocytes with round euchromatic nucleoli and sinusoidal lining. In Figure 4(b), liver tissues of diabetic rats displayed cellular abnormalities with areas of necrosis, vascular obstruction, and cellular degeneration when compared to the healthy group. The liver tissue of diabetic rats treated with extracts at a dose of 250 mg/kg indicated a slight area of cellular repair with marked vascular congestion and cellular degeneration as compared with those in the normal and diabetic control groups (Figure 4(c)), whereas in Figure 4(d), diabetic rats treated with extracts at a dose of 500 mg/kg exhibited moderate areas of cellular restoration, vascular congestion, and nuclei. The metformin group showed some typical healthy features as shown in the normal control rats with other minor degenerative changes (Figure 4(e)). Vacuolization has appeared in all the groups in different degrees, Figure 5. The highest degree was shown in the diabetic group. Treatments with 250 and 500 mg/kg aqueous extracts attenuated this effect. The percentage was down to 70% and 50%, respectively. A 200 mg/kg metformin treatment diminishes this liver damage by 30%.

**Figure 6:**
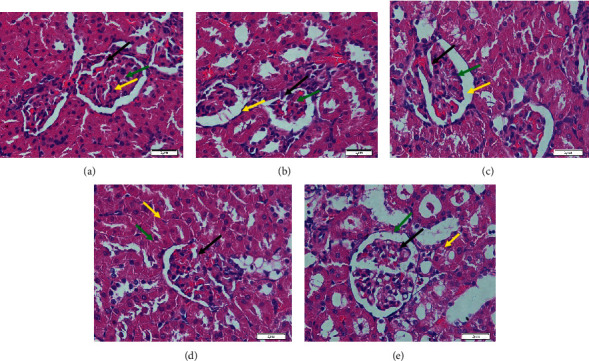
Histopathology of the kidney in (a) the normal control group; (b) the diabetic control group; (c) diabetic rats treated with 250 mg/kg extracts; (d) diabetic rats treated with 500 mg/kg extracts; and (e) diabetic rats treated with metformin 200 mg/kg, with 600 × magnification. Black arrows show glomerulus; yellow arrows show proximal tubules; and green arrows show Bowman's space.

**Figure 7:**
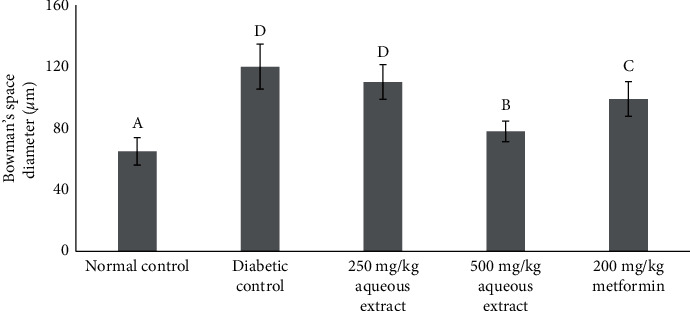
Bowman's space diameter for all groups. Different notations indicate significant differences between treatments (*p* < 0.05).

The renal glomerulus histopathology in the normal rat group (Figure 6(a)) displays the appearance of healthy kidney tissues. These were indicated by the fact that the kidney tubular structures are complete with a clear and considerable lumen and with the glomerulus and Bowmen's capsule shapes around them being clear and undamaged and coordinated with noticeable cell nuclei. Significant alterations took place in the histopathological characteristics of the kidney glomerular and tubular in the diabetic group (Figure 6(b)). The morphologic abrasions of the glomerulus have been shown with the narrowed capsule bowmen structures and glomerular necrosis. There were repair and ameliorations in the histopathological shapes in the rat treated groups, as shown in Figures 6(c), 6(d), and 6(e). Significant improvements were shown in treatment with extracts at the highest dose (500 mg/kg) and with metformin. Bowman's space and glomerulus sizes were narrowed down, and the tubule epithelium also displayed enhancement. From the calculation, Bowman's space diameter was significantly reduced with treatments of 500 mg/kg aqueous extracts and 200 mg/kg metformin, as shown in Figure 7. Importantly, 500 mg/kg aqueous extracts had a more significant effect to lower Bowman's space than metformin.

## 4. Discussion

The characterization of the secondary metabolites in the aqueous root extracts of *R. tuberosa* L. led to the detection of flavonoids, phenolics, ascorbic acids, and tannins [17]. Previously, the antioxidant activity of the aqueous root extracts of *R. tuberosa* L. has also been examined and resulted in an IC_50_ of 15.22 mg/mL that was considered as high antioxidant capacity [17]. The bioactive compounds present in this plant as shown in the FTIR study may be accountable for the hypoglycemic effect observed in this study. The FTIR spectra from the aqueous root extracts of *R. tuberosa* L. indicated the functional groups that existed were mostly phenolic and flavonoid compounds. Based on our previous research using LC-HRMS analysis [17], phenolic and flavonoid compounds were enclosed in the aqueous extract of *R. tuberosa* L.

Phenolic acids and flavonoids have been known in various studies to have high pharmacological and biological activities. These include anti-inflammatory, antioxidant, antibacterial, and antidiabetic activities [22–25]. Therefore, the presence of these phytochemicals was the importance of the study and suggestive of their application in in vivo studies [16, 26].

In this current study, we have proved for the first time the antidiabetic activities of aqueous extracts of *R. tuberosa* L. in medium-term treatments (4 weeks) and their prevention of organ complications in STZ-induced diabetic rats.

Our results indicated that the medium-term treatment with 250 and 500 mg/kg aqueous root extracts of *R. tuberosa* L. decreased considerably blood glucose in diabetic rats compared with that of untreated diabetic rats, in a dose-related manner. In addition, this treatment had lessened MDA concentrations in diabetic rats compared with those of untreated ones, also in a dose-proportional manner. This hypoglycemic activity relates to the presence of the beneficial compounds contained in the extracts, including betaine, daidzein, hispidulin, and 4-coumaric acid [17].

Daidzein and hispidulin are classified as flavonoid compounds, while 4-coumaric acid is a phenolic compound. Both phenolic and flavonoids have been suggested to have high antioxidant activity [27]. Hispidulin can act as an antidiabetic compound via stimulation of GLP‐1 secretion and suppression of hepatic glucose production [27]. Daidzein has been evaluated to have an antidiabetic activity from clinical, preclinical, and cell culture studies [28, 29]. Betaine, an oxidation product of choline, is an organic osmolyte that can protect cells from oxidative stress [30].

However, the hypoglycemic activity shown from the decrease in blood glucose and MDA pancreatic and serum concentration was not as high as when diabetic rats were treated with n-hexane [10] or hydroalcoholic root extracts [11] of *R. tuberosa* L. Furthermore, blood glucose levels in the treatment rat groups (groups 3 and 4) did not return to normal levels as in the healthy rats (group 1).

This suggests some possible explanations. Firstly, water used as a single solvent system cannot extract all secondary metabolites contained in the root of *R. tuberosa* L. Another thing is that slight decreases in blood glucose and MDA levels in the serum or rat's pancreas were due to a very large number of free radicals existing in diabetic groups. These were shown in STZ-induced diabetic rats that demonstrated highly significant hyperglycemia as compared to control healthy rats. The increase in the blood glucose and MDA levels in serum was up to 299% and 448%, respectively. Oxidative stress produced by the hyperglycemic state weakens the antioxidant defense mechanism of the body and, consequently, surges MDA levels [15]. Malondialdehyde is a product of polyunsaturated fatty acid peroxidation in cells [31]. A surge in free radicals leads to excess production of MDA. The malondialdehyde level is generally identified as an oxidative stress marker and the antioxidant status in the diabetic state [32].

It is probable that it was unable to reverse back the number of antioxidants derived from aqueous extracts to cell homeostasis or completely repair cell damages that occurred. The metformin-treated group (group 5) also showed a substantial reduction in their blood glucose and MDA levels. Nonetheless, their decreases did not successfully reverse back blood glucose and MDA concentrations to normal conditions in the same way as in the normal control group (group 1).

Similar trends were also shown in the histopathological alterations in the diabetic rats' organs. Treatment with the aqueous root extracts of *R*. *tuberosa* L. at a 250 mg/kg dose cannot restore pathological changes of the pancreas to a healthy level, while a 500 mg/kg dose showed a moderate restoration to the healthy condition. Although, the Langerhans islet diameter was significantly elevated in the treated rats in the 500 mg/kg dose compared to that of the diabetic group. The observed nephroprotective effects of the aqueous root extracts in terms of normalizing the glomerular size, the number of proximal tubular cells, and Bowman's space diameter were also shown in the treatment with the highest dose (500 mg/kg). Nonetheless, histopathological examination of the liver revealed a significant number of repair changes in treated diabetic animals compared to the untreated ones, from the treatment dose of 250 mg/kg. This was shown in the significant decline in the vacuolization degrees.

Treatments with aqueous root extracts significantly improve pathological changes of the pancreas, liver, and kidney most probably due to their high flavonoid and phenolic content. Polyphenol compounds are suggested to have significant effects on the regulation of oxidative stress through a number of mechanisms. These include reducing inflammation through inactivation of mitogen-activated protein kinase (MAPK) signalling pathways [33], repairing DNA damages caused by oxidative stress via the nuclear factor erythroid 2-related factor 2 (Nrf2) pathway [34], and decreasing apoptosis through regulating protein kinase B (AKT)/caspase expression [35].

We believe that this is the first study on the in vivo pharmacological evaluation of this antidiabetic effect of the aqueous root extracts of *R. tuberosa* L. Yet, it is suggested that a higher dose of treatments, more than 500 mg/kg, can be applied to achieve more prominent effects. Besides, the root extracts of *R. tuberosa* L. can also be extracted with water with an addition of acid to attract more secondary metabolites.

## 5. Conclusions

In conclusion, our current study has established that the aqueous root extracts of *R. tuberosa* L. could be considered to be used as antidiabetic drugs. The antihyperglycemic activity is suggested from the significant reduction in blood glucose levels, MDA serum levels, and pancreatic levels after medium-term administration of the aqueous root extracts of *R. tuberosa* L. Additionally, the aqueous root extracts of *R. tuberosa* L. have shown a crucial preventive effect against pancreas, renal fibrosis, and liver complications. Nevertheless, a higher dose of aqueous extracts is suggested to attain more substantial outcomes.

## Figures and Tables

**Table 1:** Blood glucose levels on rats at the end of the treatment, in week 4, in groups 1 to 5.

Group	Blood glucose level (mg/dL)^*∗*^	Changes (%)
1 (normal)	131.2 ± 18.12a	
2 (diabetic)	523.8 ± 17.99e	299.24
3 (dose of 250 mg/kg)	454.6 ± 12.52d	13.21
4 (dose of 500 mg/kg)	391.2 ± 17.44c	25.35
5 (metformin 200 mg/kg)	165.4 ± 13.55ab	68.42

*∗*Different notations indicate significant differences between treatments (*p* < 0.05).

**Table 2:** Serum and pancreas MDA levels on rats at the end of the treatment, in week 4, in groups 1 to 5.

Group	Serum MDA level (*μ*g/dL)*∗*	Changes	Pancreas MDA level (*μ*g/dL)*∗*	Changes
1 (normal)	1.27 ± 0.18a		0.237 ± 0.03a	
2 (diabetic)	6.96 ± 0.41e	448.03%	0.75 ± 0.12 d	216.45%
3 (dose of 250 mg/kg)	6.04 ± 0.15 d	13.22%	0.69 ± 0.09 d	8.0%
4 (dose of 500 mg/kg)	4.55 ± 0.33c	34.63%	0.66 ± 0.07c	12.0%
5 (metformin 200 mg/kg)	2.35 ± 0.45 b	66.24	0.367 ± 0.03 b	51.07%

*∗*Different notations indicate significant differences between treatments (*p* < 0.05).

## Data Availability

The data used to support this study are included in the article and will be made available on request.
